# Fatigue Resistance and Mitochondrial Adaptations to Isometric Interval Training in Dystrophin‐Deficient Muscle: Role of Contractile Load

**DOI:** 10.1096/fj.202500618RR

**Published:** 2025-05-14

**Authors:** Nao Yamauchi, Yuki Ashida, Azuma Naito, Nao Tokuda, Ayaka Niibori, Norio Motohashi, Yoshitsugu Aoki, Takashi Yamada

**Affiliations:** ^1^ Graduate School of Health Sciences Sapporo Medical University Sapporo Japan; ^2^ The Japan Society for the Promotion of Science (JSPS) Tokyo Japan; ^3^ Department of Molecular Therapy National Institute of Neuroscience, National Center of Neurology and Psychiatry Tokyo Japan; ^4^ Graduate School of Biomedical and Health Sciences Hiroshima University Hiroshima Japan

**Keywords:** contractile load, fatigue resistance, isometric interval training, mitochondria, muscular dystrophy

## Abstract

In normal mouse skeletal muscles, interval training (IT)‐mimicking neuromuscular electrical stimulation enhances muscle fatigue resistance and mitochondrial content, with greater gains observed at high (100 Hz stimulation, IT100) compared to low (20 Hz stimulation, IT20) contractile load. In this study, we compared the effects of repeated IT100 and IT20 on fatigue resistance and mitochondrial adaptations in young male mdx52 mice (4‐ to 6‐week‐old), an animal model for Duchenne muscular dystrophy. Plantar flexor muscles were stimulated in vivo using supramaximal electrical stimulation to induce isometric contractions every other day for 4 weeks (a total of 15 sessions). In non‐trained muscles of mdx52 mice, decreased fatigue resistance was associated with reduced citrate synthase activity, lower peroxisome proliferator‐activated receptor γ coactivator 1 alpha (PGC‐1α) protein expression, and diminished levels of mitochondrial respiratory chain complex II, and an increased percentage of Evans Blue dye‐positive areas. IT100, but not IT20, markedly improved fatigue resistance and restored all these alterations in mdx52 mice. Furthermore, an acute session of IT100, but not IT20, led to increased phosphorylation of p38 mitogen‐activated protein kinase (MAPK) and elevated mRNA levels of PGC‐1α, which were blocked by the p38 MAPK inhibitor SB203580. These findings suggest that contractile load is a key determinant of isometric IT‐induced improvements in fatigue resistance, even in dystrophin‐deficient muscles, potentially through a p38 MAPK/PGC‐1α‐mediated increase in mitochondrial content.

## Graphical abstract

High‐load, but not low‐load, isometric interval training (IT) increased mitochondrial content and muscle fatigue resistance in mdx52 mice, a model for Duchenne muscular dystrophy. High‐load isometric IT restored citrate synthase activity, peroxisome proliferator‐activated receptor γ coactivator 1 alpha (PGC‐1α) expression, and mitochondrial complex II levels, while reducing muscle damage. These effects were linked to transient activation of the p38 mitogen‐activated protein kinase (MAPK)/PGC‐1α pathway, which was blocked by the p38 MAPK inhibitor SB203580. Contractile load is a key determinant of isometric IT‐induced increases in fatigue resistance in dystrophic muscle.

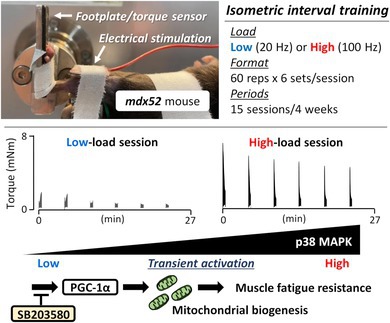

**Abbreviations**
AMPK: AMP‐activated protein kinase
CS: citrate synthase
DMD: Duchenne muscular dystrophy
EBD: Evans Blue dye
ERK1/2: extracellular signal‐regulated kinases 1 and 2
IT: interval training
JNK: *c‐Jun* N‐terminal kinases
MAPK: mitogen‐activated protein kinase
MyHC: myosin heavy chain
PGC‐1α: peroxisome proliferator‐activated receptor γ coactivator 1 alpha
WT: wild‐type
α‐SG: α‐sarcoglycan
β‐DG: β‐dystroglycan

## 1 Introduction

Duchenne muscular dystrophy (DMD) is a progressive, X‐linked muscle degenerative disorder characterized by the absence of dystrophin protein. Patients with DMD [1] and mdx mice [2], a commonly used model for DMD, experience elevated muscle fatigability. Impaired aerobic capacity due to mitochondrial dysfunction has been implicated as a primary cause of reduced fatigue resistance in muscles lacking dystrophin [3, 4], although other factors may also contribute [5].

Conversely, exercise is a potent trigger for mitochondrial adaptation and can enhance fatigue resistance. However, susceptibility to damage induced by eccentric contractions is increased in dystrophin‐deficient muscles [6], which has led to the avoidance of aggressive exercise prescriptions [7, 8, 9, 10]. Nevertheless, recent studies have provided evidence that training with isometric contractions, which impose relatively low levels of damage [11], improves muscle function in both DMD patients [12] and experimental animal models [13, 14].

Interval training (IT) with high‐intensity contractions is emerging as a promising method for enhancing fatigue resistance in both healthy individuals and those with certain diseases [15]. Previously, we demonstrated that IT‐mimicking neuromuscular electrical stimulation, delivered as isometric contractions, induces greater mitochondrial adaptations and improves fatigue resistance at higher intensities compared to lower intensities in normal mice [16]. Based on these findings, we recently reported that isometric IT with high contractile load increases mitochondrial content and fatigue resistance, along with amelioration of dystrophic pathology, in 15‐ to 22‐week‐old exon 52‐deficient X chromosome‐linked muscular dystrophy (mdx52) mice [14]. However, it remains unclear whether isometric IT is safe and effective in improving muscle fatigue resistance in young mdx52 mice undergoing vigorous degeneration and regeneration [17, 18], or whether the beneficial effects of isometric IT are contractile load‐dependent even in the dystrophic muscles.

Peroxisome proliferator‐activated receptor gamma coactivator 1 alpha (PGC‐1α) is a key regulator of exercise‐induced mitochondrial biogenesis and cellular respiration [19]. Notably, PGC‐1α gene transfer has been demonstrated to enhance mitochondrial biogenesis and fatigue resistance in the muscle of mdx mice [20, 21]. Moreover, mitogen‐activated protein kinases (MAPKs) are a conserved family of serine/threonine protein kinases, primarily consisting of p38 MAPK, extracellular signal‐regulated kinases 1 and 2 (ERK1/2), and *c‐Jun* N‐terminal kinases (JNK) [22]. Among them, the p38 MAPK signaling pathway is a well‐established regulator of PGC‐1α expression in skeletal muscle [23]. Interestingly, the p38 MAPK/PGC‐1α pathway has been shown to be activated in an intensity‐dependent manner following exercise in normal muscles [24].

Although exercise‐induced transient activation of MAPK triggers various beneficial adaptations in normal skeletal muscle, including enhanced substrate metabolism capacity [25], persistent activation of these signaling pathways has been associated with the progression of various pathologies [26]. Indeed, prolonged treadmill exercise, which involves eccentric contractions during movement [27], has been shown to induce chronic MAPK activation linked to the exacerbation of tissue pathology in dystrophin‐deficient muscles [28].

Therefore, in the present study, we tested the following hypotheses: (i) Electrical stimulation‐induced isometric IT, which is a safer contraction mode in dystrophic muscle [11], would lead to greater improvements in muscular fatigue resistance and mitochondrial adaptations with high contractile load compared to low contractile load, even in young mdx52 mice, where degeneration and regeneration occur extensively; and (ii) These beneficial adaptations are associated with intermittent activation of the p38 MAPK/PGC‐1α pathway.

## 2 Materials and Methods

### 2.1 Ethical Approval

Male C57BL/6J wild‐type (WT) mice were obtained from the Jackson Laboratory Japan (Kanagawa, Japan). The mdx52 mice were generated using a gene‐targeting approach [29] and had been backcrossed to the WT strain for over 10 generations. These mdx52 mice were selected due to the location of their deletion, which corresponds to the hotspot region between exons 45 and 55, where approximately 70% of DMD patients have deletion mutations [29, 30]. The mice were acclimated for at least 3 days prior to experimentation. They were housed on a standard chow diet with access to water and maintained on a 12 h light–dark cycle.

All mouse experiments were performed at Sapporo Medical University (Sapporo, Japan) in accordance with approved protocols from the Committee on Animal Experiments of Sapporo Medical University (No. 20‐084, 21‐062, and 21‐103). Animal care adhered to institutional guidelines. For in situ muscle experiments, mice were anesthetized with 2% inhaled isoflurane until a stable anesthetic plane was achieved, with a consistent breathing rate and no response to a toe pinch. At the end of the experiment, mice were euthanized by rapid cervical dislocation under isoflurane anesthesia, after which the muscles were isolated. A total of 68 mice were used.

### 2.2 Experimental Design

**FIGURE 1:**
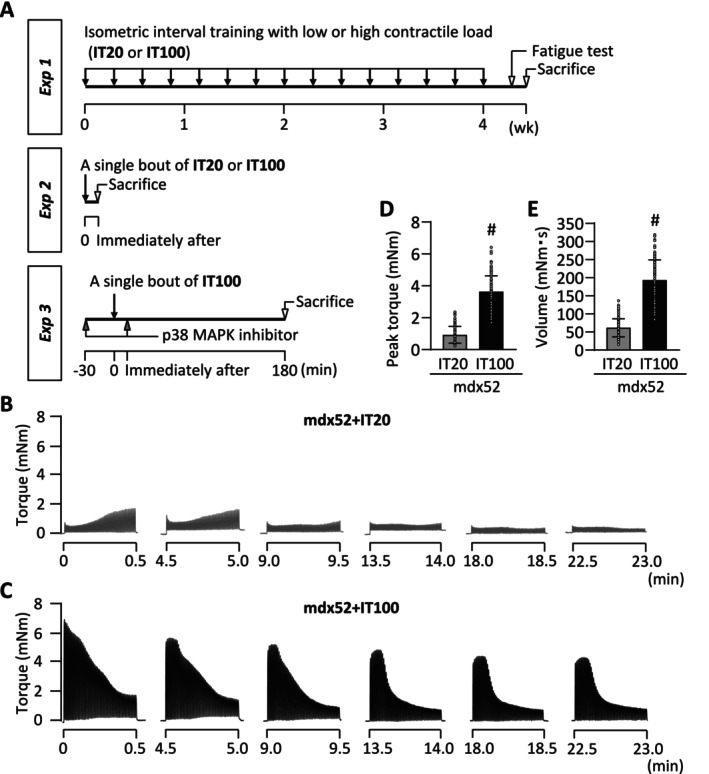
Schematic overview of the experimental design. (A) In Experiment (Exp) 1, fatigue resistance and intracellular events were evaluated in WT and mdx52 mice with and without interval training (IT) at low contractile load (20 Hz stimulation, IT20) or high contractile load (100 Hz stimulation, IT100). IT was performed using electrical stimulation every other day for a total of 15 sessions. In Exp 2, cellular signaling underlying IT‐induced physiological adaptations was investigated after an acute single bout of IT20 or IT100 in mdx52 mice. In Exp 3, the mdx52 mice underwent a single bout of IT100 with or without the administration of the p38 MAPK inhibitor SB203580. Typical torque traces during an IT20 (B) and IT100 (C) session. The mean peak torque (D; *n* = 720 per group) and torque‐time integral (volume) (E, *n* = 120 per group) were calculated across all training sets and sessions for all mice. Data are presented as individual values and mean ± SD. Unpaired *t*‐test was performed. ^#^ *p* < 0.05 versus WT.

To investigate the role of contractile load in isometric IT‐induced molecular and physiological adaptations in dystrophin‐deficient muscles, we conducted three separate experiments (Figure 1A).

#### 2.2.1 Experiment 1

We examined the effect of isometric IT with different contraction intensities on muscle fatigability and mitochondrial content in mdx52 mice. Four‐ to six‐week‐old male C57BL/6J WT mice (*n* = 8) and mdx52 mice (*n* = 24) were used. The mdx52 mice were randomly assigned to three groups: a non‐trained control group (mdx52: *n* = 8), a low contractile load IT group (*n* = 8), and a high contractile load IT group (*n* = 8). The IT protocol, with either low or high contractile load, was performed on the left leg every other day for 4 weeks (a total of 15 sessions). The force generated by electrical stimulation can be adjusted by changing the stimulation frequency, as the relationship between force and frequency is sigmoidal [31]. Thus, in accordance with our previous study using normal mice [16], IT with low or high contractile load was applied to mdx52 mice using repeated electrical stimulation at 20 and 100 Hz, respectively, and hence the groups were referred to as mdx52+IT20 and mdx52+IT100.

Throughout each IT session, mice were anesthetized with isoflurane inhalation. They were placed in a supine position on a platform, with the foot secured to a footplate connected to a torque sensor (S‐14154, Takei Scientific Instruments) at an angle of 0° dorsiflexion (i.e., 90° relative to the tibia). The plantar flexor muscles were activated by supramaximal (45 V, 0.5 ms) monophasic rectangular current pulses delivered via a pair of surface electrodes to evoke isometric contractions. A previous study demonstrated that this method recruits all fibers in the plantar flexor muscles [32]. The stimulation protocol was specifically designed to mimic IT based on maximal cycling efforts, consisting of 0.25 s tetanic contractions every 0.5 s [16, 33, 34, 35]. Each session comprised six sets of 60 contractions, with 4 min intervals between sets. The mean peak torque and torque‐time integral (volume) were calculated across all training sets and sessions for all mice.

Forty‐eight hours after the final IT session, in vivo fatigue resistance of the plantar flexor muscles was measured in each group using 50 repeated 350 ms, 70 Hz tetani given at 3 s intervals. The conditions of the fatigue test were determined based on two considerations: (1) previous research has demonstrated that the most commonly occurring firing frequency of motor units during locomotion is approximately 70 Hz in rodent fast‐twitch muscles [36], and (2) to facilitate direct comparison with our prior studies [14, 16, 34, 35], which employed the same training protocol and fatigue test. Twenty‐four hours after measuring fatigue resistance (i.e., 72 h after the final IT session), the mice were euthanized by cervical dislocation under isoflurane anesthesia, and the plantar flexor muscles were excised from each animal. The plantaris muscles were snap‐frozen in liquid nitrogen and stored at −80°C for later analysis (i.e., citrate synthase [CS] activity). For histological analysis, the mid‐belly of the medial gastrocnemius muscles was frozen in pre‐cooled isopentane and stored at −80°C. The remaining portions of the medial and lateral gastrocnemius muscles were quickly minced with scissors at 4°C and stored at −80°C for later analysis (i.e., myosin heavy chain (MyHC) isoforms and immunoblots).

#### 2.2.2 Experiment 2

We conducted Experiment 2 to measure cellular signaling for oxidative adaptation after the first IT session, as the activation of these signaling molecules is temporary [24], making it necessary to collect muscle samples immediately after the IT session. Male C57BL/6J WT (*n* = 6) and mdx52 mice (*n* = 18), aged 6‐ to 8‐week‐old, were divided into the same groups as in Experiment 1 (i.e., WT, mdx52, mdx52+IT20, mdx52+IT100). Immediately after one IT20 or IT100 session, the mice were euthanized by rapid cervical dislocation under isoflurane anesthesia, and the muscles were then isolated. The levels of phosphorylation for p38 MAPK Thr180/Tyr182, ERK1/2 Thr202/Tyr204, JNK Thr183/Tyr185, and AMP‐activated protein kinase (AMPK) α Thr172 were examined in the gastrocnemius muscles from each animal.

#### 2.2.3 Experiment 3

Based on the results of Experiments 1 and 2, muscle fatigue resistance was increased in mdx52 mice following IT100, but not IT20, which was accompanied by enhanced phosphorylation of p38 MAPK and elevated expression of PGC‐1α. Therefore, another set of experiments was conducted to examine the role of p38 MAPK in IT100‐induced PGC‐1α expression in dystrophin‐deficient muscles. Seven‐week‐old male mdx52 mice (*n* = 12) underwent a single session of IT100 with (*n* = 6) or without (*n* = 6) the administration of SB203580, a p38 MAPK inhibitor. SB203580 (25 mg/kg) was administered intraperitoneally 30 min before and immediately after the IT100 session [37]. The phosphorylation level of p38 MAPK Thr180/Tyr182 and the mRNA expression levels of PGC‐1α were measured 180 min after the IT100 session.

### 2.3 Myosin Heavy Chain Isoforms Separation

The gastrocnemius muscles were homogenized in ice‐cold homogenization buffer (30 μL/mg wet wt) containing (mM): Tris maleate, 10; NaF, 35; NaVO_4_, 1; 1% Triton X‐100 (vol/vol), and 1 tablet of protease inhibitor cocktail (Roche) per 50 mL. Protein concentration was measured using the Bradford assay [38]. Aliquots of the whole muscle homogenates (5 μg) were mixed with SDS sample buffer containing (mM): Tris/HCl, 62.5; 2% SDS (wt/vol); 10% glycerol (vol/vol); 5% 2‐mercaptoethanol (vol/vol); and 0.02% bromophenol blue (wt/vol). Proteins were then loaded onto a 6.8% polyacrylamide slab gel [39]. Electrophoresis was performed at 4°C for 24 h at 160 V, followed by staining with Coomassie brilliant blue. Gel images were analyzed densitometrically using ImageJ.

### 2.4 Histopathological Analyses

Tissue sections (10 μm) from the mid‐belly of the medial gastrocnemius muscles were stained with hematoxylin and eosin. For Evans Blue dye (EBD) quantification, a 1% (wt/vol) EBD solution (1 mg/10 g body wt) was intraperitoneally injected under isoflurane anesthesia 9 h before euthanasia [32]. Images of the entire section were captured using a BZ‐X700 microscope (KEYENCE, Osaka, Japan) with a 4× objective lens. The images were then stitched together using BZ‐X700 analyzer software (KEYENCE) and exported as TIFF files. The percentage of the EBD‐positive area relative to the total cross‐sectional muscle area in a single section of each muscle was quantified using the BZ‐X700 analyzer software.

### 2.5 Mitochondrial Enzyme Activity

CS activity is frequently used as an indicator of mitochondrial content, although transmission electron microscopy remains the gold standard for accuracy [40]. Maximal CS activity was assessed in plantaris muscle homogenates. Briefly, the muscles were homogenized in ice‐cold 100 mM potassium phosphate buffer (100 μL/mg wet wt) and maximal CS activity was measured spectrophotometrically as described previously [41].

### 2.6 Immunoblots

Immunoblots were performed using the following antibodies: anti‐utrophin (kindly provide by Dr. Michihiro Imamura, National Center of Neurology and Psychiatry [NCNP]) [42], anti‐β‐dystroglycan (β‐DG, kindly provide by Dr. Michihiro Imamura, NCNP) [43], anti‐α‐sarcoglycan (α‐SG, kindly provide by Dr. Michihiro Imamura, NCNP) [44], anti‐integrin α7B (kindly provide by Dr. Eva Engvall, Burnham Institute) [45], anti‐integrin β1D (kindly provide by Dr. Eva Engvall) [45], anti‐dysferlin (VP‐D503, Vector), anti‐PGC‐1α (ab54481, Abcam), anti‐total OXPHOS rodent WB antibody cocktail (ab110413, Abcam), anti‐phospho‐p38 MAPK Thr180/Tyr182 (#4511, Cell signaling), anti‐p38 MAPK (#9212, Cell signaling), anti‐phospho‐ERK1/2 Thr202/Tyr204 (#9101, Cell signaling), anti‐ERK1/2 (#9102, Cell signaling), anti‐phospho‐JNK Thr183/Tyr185 (#9251, Cell signaling), anti‐JNK (#9252, Cell signaling), anti‐phospho‐AMPKα Thr172 (#2531, Cell signaling), anti‐AMPKα (#2532, Cell signaling).

The gastrocnemius muscle homogenates, as described in MyHC separation, were used to measure the expression levels of utrophin, β‐dystroglycan, α‐sarcoglycan, integrin α7B, integrin β1D, and dysferlin. On the other hand, muscle homogenates were centrifuged at 14 000*g* for 15 min at 4°C, and the supernatant, representing the soluble fraction, was used to measure the expression levels of PGC‐1α, OXPHOS, phospho/total p38 MAPK, phospho/total ERK1/2, phospho/total JNK, phospho/total AMPKα. The protein content was quantified using the Bradford assay [38]. Aliquots of either whole or soluble proteins (5 μg) were mixed with SDS sample buffer. The proteins were loaded onto a 4%–15% Criterion Stain‐Free gel (BioRad, Hercules, CA). After imaging the gels using a BioRad Stain‐Free imager, proteins were transferred to polyvinylidine fluoride membranes. The membranes were blocked with 3% (wt/vol) nonfat milk in Tris‐buffered saline containing 0.05% (vol/vol) Tween 20, followed by overnight incubation at 4°C with primary antibodies. Membranes were then washed and incubated for 1 h at room temperature with secondary antibodies (1:10 000, donkey‐anti‐rabbit or donkey‐anti‐mouse, BioRad). After exposure to a chemiluminescence substrate (Millipore), membrane images were captured using a charge‐coupled device camera connected to the ChemiDOC MP system (BioRad), and densitometric analysis was performed using Image Lab Software (BioRad).

### 2.7 Quantitative Real‐Time PCR

Real‐time PCR was used to quantify the mRNA levels of PGC‐1α in frozen gastrocnemius muscle tissue. Briefly, total RNA was extracted using TRIzol reagent (Invitrogen, Carlsbad, CA), and the purity and yield of the extracted RNA were assessed by measuring absorbance at 260 and 280 nm with a Nanodrop Light spectrophotometer (Thermo Scientific, Waltham, MA). Total RNA was then reverse‐transcribed into cDNA using the PrimeScript RT Reagent Kit (Takara, Japan). The cDNA products were analyzed using a StepOne Real‐Time PCR System (Applied Biosystems, Foster City, CA, USA) with the SYBR Green PCR Master Mix protocol.

The specific primers used in this study are as follows: PGC‐1α forward primer 5′‐CGG AAA TCA TAT CCA ACC AG‐3′, PGC‐1α reverse primer 5′‐TGA GGA CCG CTA GCA AGT TTG‐3′ [46]; 36B4 forward primer 5′‐GGC CCT GCA CTC TCG CTT TC‐3′, 36B4 reverse primer 5′‐TGC CAG GAC GCG CTT GT‐3′ [14]. The oligonucleotides were purchased from FASMAC Co. Ltd. (Kanagawa, Japan). All samples were run in duplicate. Relative amounts of target mRNA were determined using the comparative threshold cycle method (ΔΔCT). Expression of target genes was normalized to the expression level of 36B4.

### 2.8 Statistics

Data are presented as mean ± SD. Data normality was examined with the Shapiro–Wilk test. In Experiment 1, for normally distributed data (maximum absolute torque, CS activity, the expression levels of utrophin, integrin α7B, integrin β1D, β‐DG, α‐SG, dysferlin, PGC‐1α, NDUFB8, SDHB, UQCRC, MTC01, ATP5, and the phosphorylation levels of p38 MAPK Thr180/Tyr182, ERK1/2 Thr202/Tyr204, JNK Thr183/Tyr185 relative to total protein content), one‐way ANOVA was used to determine the mean differences among the four groups (WT, mdx52, mdx52+IT20, and mdx52+IT100 group). If data exhibited a non‐normal distribution (EBD positive area and the distribution of the MyHC isoforms), a Kruskal–Wallis one‐way ANOVA was used on ranks. Fatigue resistance (group × repetitions) was assessed by two‐way repeated‐measures ANOVA. In Experiment 2, for normally distributed data (the phosphorylation levels of p38 MAPK Thr180/Tyr182, JNK Thr183/Tyr185, and AMPK Thr172), one‐way ANOVA was used to determine the mean differences between the groups. If data exhibited a non‐normal distribution (ERK1/2 Thr202/Tyr204), a Kruskal–Wallis one‐way ANOVA was used on ranks. In Experiment 3, two‐way ANOVA was used to determine the mean differences between the groups (exercise × inhibitor). When these ANOVA tests showed significance, a Tukey post hoc test was performed. A *p* value < 0.05 was regarded as statistically significant. A power test was performed assuming that IT induces a 35% ± 15% change in physiological measurements relative to the control value [14]. With a power of 0.80 and an alpha of 0.05, this gives a sample size of six. Based on this, we used six to eight animals in each group in all experiments. Statistical testing was performed with SigmaPlot (version 13, Systat Software Inc.).

## 3 Results

### 3.1 Isometric Interval Training With High, but Not Low, Contractile Load Improves Fatigue Resistance in Dystrophin‐Deficient Muscle

Typical torque traces during IT20 and IT100 sessions in mdx52 mice are shown in Figure 1B,C, respectively. The averaged peak torque and torque‐time integral (volume) across all training sets and sessions were 4 and 3.2 times higher in the IT100 group than in the IT20 group, respectively (3.6 ± 1.2 mNm vs. 0.9 ± 0.6 mNm, *p* < 0.001; 193 ± 56 vs. 61 ± 25 mN·m·s, < 0.001) (Figure 1D,E).

**FIGURE 2:**
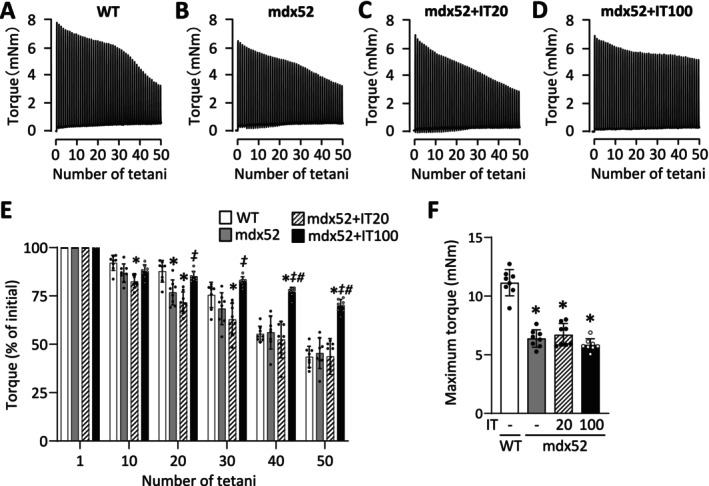
Isometric interval training with high, but not low, contractile load improves fatigue resistance in dystrophin deficient muscle. (A–D) Representative torque recording during the in vivo fatigue protocol (70 Hz, 350 ms tetani every 3 s) of the plantar flexor muscles from WT and mdx52 mice. The mdx52 mouse underwent isometric interval training (IT) with either low (IT20) or high (IT100) contractile load. (E) Mean (±SD) relative tetanic torque during fatiguing stimulation. The torque in the first tetanus was set to 100% for each muscle. Data are presented as mean ± SD for eight muscles per group. A two‐way repeated measures ANOVA with Tukey post hoc test was performed. *p < 0.05 versus WT, ^#^ *p* < 0.05 versus mdx52, ^‡^ *p* < 0.05 versus mdx52+IT20. (F) Maximum isometric torque. Data are presented as mean ± SD for eight muscles per group. A Kruskal–Wallis one‐way ANOVA was used on ranks. *p < 0.05 versus WT.

Figure 2A–D displays typical torque records during in vivo fatiguing stimulations of the plantar flexor muscles from all experimental groups. The percentage of initial torque at the 20th tetanus during the fatigue protocol was significantly lower in the mdx52 group compared to the WT group (*p* = 0.026) (Figure 2E), indicating that the mdx52 muscles have less fatigue resistance than normal muscles. Similarly, in the mdx52+IT20 group, the percentage of initial torque during the fatigue protocol was lower at the 10th, 20th, and 30th tetani compared to the WT group (10th, *p* = 0.045; 20th, *p* < 0.001; 30th, *p* = 0.004). In contrast, the percentage of initial torque in the mdx52+IT100 group was higher than in the mdx52 group at the 40th and 50th tetani (40th, *p* < 0.001; 50th, *p* < 0.001), and it was also higher than in the mdx52+IT20 group after the 20th tetanus (20th, *p* = 0.026; 30th, *p* < 0.001; 40th, *p* < 0.001; 50th, *p* < 0.001).

In accordance with our previous study [47], the maximum isometric torque was significantly lower in the mdx52 group than in the WT group (WT [*n* = 8] 11.1 ± 1.1 mNm vs. mdx52 [*n* = 8] 6.4 ± 0.7 mNm, *p* < 0.001) (Figure 2F). In contrast to fatigue resistance, neither IT20 nor IT100 altered the maximum isometric torque in mdx52 mice ([mdx52+IT20] 6.7 ± 1.0 mNm, *p* = 0.879 vs. mdx52; [mdx52+IT100] 5.8 ± 0.5 mNm, *p* = 0.599 vs. mdx52).

### 3.2 Isometric Interval Training With High, but Not Low, Contractile Load Abolishes EBD‐Positive Fibers in Dystrophin‐Deficient Muscles

**FIGURE 3:**
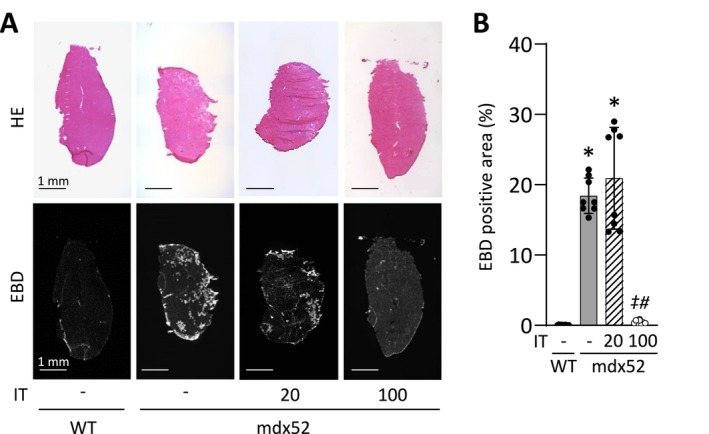
Isometric interval training with high, but not low, contractile load abolishes EBD positive fibers in dystrophin‐deficient muscles. Representative images of transverse sections of medial gastrocnemius muscles stained for hematoxylin and eosin (H&E) and Evans Blue dye (EBD) of gastrocnemius muscles in WT and mdx52 mice. The mdx52 mouse underwent isometric interval training with either high (IT100) or low contractile load (IT20) (A). Scale bars 1 mm. The percentage of the EBD‐positive area over the total cross‐sectional muscle area (B). Data show mean ± SD for eight muscles per group. A Kruskal–Wallis one‐way ANOVA was used on ranks. *p < 0.05 versus WT, ^#^ *p* < 0.05 versus mdx52, ^‡^ *p* < 0.05 versus mdx52+IT20.

In the mdx group, 18% of muscle fibers were positively stained by EBD, indicating increased membrane permeability [48] (Figure 3A,B). Remarkably, these damaged fibers were almost completely eliminated by IT100 (0.5% ± 0.3%, *p* = 0.048 vs. mdx52), but not by IT20 (21.1% ± 7.4%, *p* = 1.000 vs. mdx52).

**FIGURE 4:**
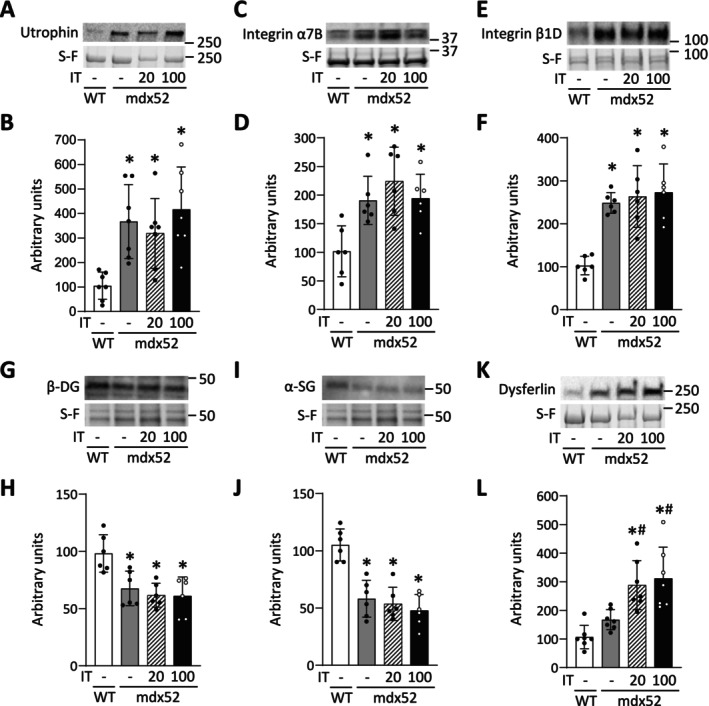
Effects of isometric interval training with high, but not low, contractile load on the levels of proteins involved in membrane integrity and repair. Representative Stain‐Free (S‐F) images and western blots illustrating the levels of utrophin (A), integrin α7B (C), integrin β1D (E), β‐dystroglycan (DG) (G), α‐sarcoglycan (SG) (I), and dysferlin (K) in gastrocnemius muscles in WT and mdx52 mice. The mdx52 mouse underwent isometric interval training with either high (IT100) or low contractile load (IT20). The levels of utrophin (B), integrin α7B (D), integrin β1D (F), β‐DG (H), α‐SG (J), and dysferlin (L) were normalized to total protein in S‐F images. Data show mean ± SD for 6–eight muscles per group. One‐way ANOVA with Tukey post hoc test was performed. *p < 0.05 versus WT, ^*#*^ *p* < 0.05 versus mdx52.

To investigate the mechanism behind the reduction of resting muscle damage caused by IT100, we examined proteins involved in membrane integrity (i.e., utrophin, integrin α7B, integrin β1D, β‐DG, α‐SG) and repair (i.e., dysferlin). Compared to the WT group, the levels of utrophin, integrin α7B, and integrin β1D were increased in the mdx52, mdx52+IT20, and mdx52+IT100 groups ([utrophin] *p* = 0.008, *p* = 0.037, and *p* = 0.002, respectively; [integrin α7B] *p* = 0.020, *p* = 0.001, and *p* = 0.015, respectively; [integrin β1D] *p* < 0.001, *p* < 0.001, and *p* < 0.001, respectively), with no differences between those groups (Figure 4A–F). Conversely, the expression levels of β‐DG and α‐SG were significantly lower in the mdx52, mdx52+IT20, and mdx52+IT100 groups compared to the WT group ([β‐DG] *p* = 0.010, *p* = 0.002, and *p* = 0.002, respectively; [α‐SG] *p* < 0.001, *p* < 0.001, and *p* < 0.001, respectively) (Figure 4G–J). There was no difference in the amount of dysferlin between the WT and mdx52 groups (*p* = 0.441). However, the expression of dysferlin was similarly increased in both the mdx52+IT20 and mdx52+IT100 groups compared to the mdx52 groups (*p* = 0.027 and *p* = 0.007, respectively) (Figure 4K,L).

### 3.3 Isometric Interval Training With High, but Not Low, Contractile Load Increases Mitochondrial Content and Respiratory Complexes in Dystrophin‐Deficient Muscles

**FIGURE 5:**
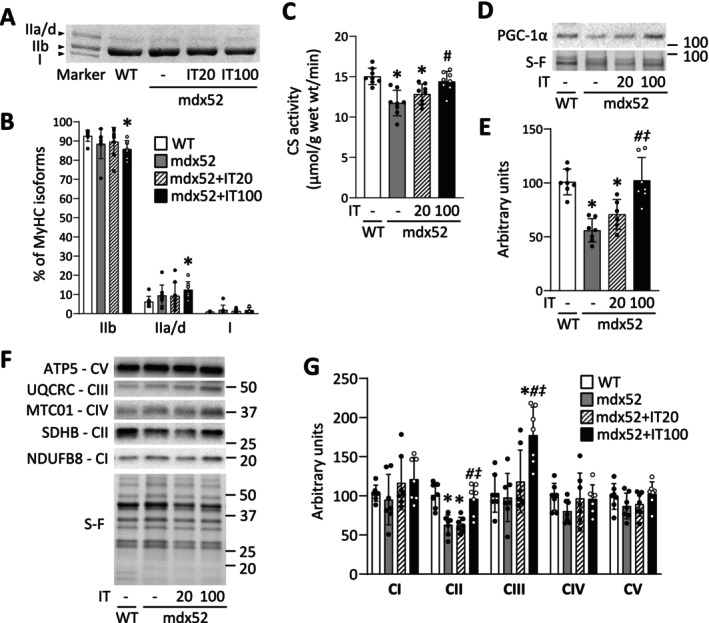
Isometric interval training with high, but not low, contractile load increases mitochondrial content and respiratory complexes in dystrophin‐deficient muscles. (A) Blots showing electrophoretically separated myosin heavy chain (MyHC) isoforms in the gastrocnemius muscles of each group. (B) Distribution of MyHC isoforms. Citrate synthase (CS) activity (C) in plantaris muscles from WT and mdx52 mice. The mdx52 mouse underwent isometric interval training with either high (IT100) or low contractile load (IT20). Representative Stain‐Free (S‐F) images and western blots illustrating the levels of peroxisome proliferator activated receptor γ coactivator 1 alpha (PGC‐1α) (D), complex I (CI) subunit (NDUFB8), CII subunit (SDHB), CIII subunit (UQCRC), CIV subunit (MTC01), CV subunit (ATP5) (F) in gastrocnemius muscles. The levels of PGC‐1α (E) and CI‐V (G) were normalized to total protein in S‐F images. Data show mean ± SD for six to eight muscles per group. One‐way ANOVA with Tukey post hoc test was performed, except for the distribution of the MyHC isoforms, where a Kruskal–Wallis one‐way ANOVA was used on ranks. *p < 0.05 versus WT, ^#^ *p* < 0.05 versus mdx52, ^‡^ *p* < 0.05 versus mdx52+IT20.

The distribution of MyHC isoforms did not differ between the WT, mdx52, and mdx52+IT20 groups ([WT vs. mdx52] IIb, *p* = 0.395; IIa/d, *p* = 0.349; I, *p* = 0.359 [WT vs. mdx52+IT20] IIb, *p* = 0.787; IIa/d, *p* = 0.710; I, *p* = 0.359 [mdx52 vs. mdx52+IT20] IIb, *p* = 0.919; IIa/d, *p* = 0.936; I, *p* = 0.359) (Figure 5A,B). On the other hand, compared to the WT group, the mdx52+IT100 group showed a decrease in the proportion of MyHCIIb (*p* = 0.018) and an increase in MyHCIIa/d (*p* = 0.024). However, MyHC composition did not differ between the mdx52+IT100 group and either the mdx52 or mdx52+IT20 group ([mdx52 vs. mdx52+IT100] IIb, *p* = 0.525; IIa/d, *p* = 0.644; I, *p* = 0.359 [mdx52+IT20 vs. mdx52+IT100] IIb, *p* = 0.188; IIa/d, *p* = 0.293; I, *p* = 0.359). These data suggest that the changes in fatigue resistance due to dystrophin deficiency and IT100 are independent of muscle fiber type transition.

In contrast, compared to the WT group, mdx52 mice exhibited reduced CS activity, an indicator of mitochondrial content [40] (*p* < 0.001), and lower levels of PGC‐1α, a key regulator of mitochondrial biogenesis [19] (*p* < 0.001) (Figure 5C–E). IT100, but not IT20, restored these negative alterations ([CS activity] *p* = 0.002 and *p* = 0.320, respectively; [PGC‐1α] *p* < 0.001 and *p* = 0.316, respectively). Furthermore, the amount of mitochondrial respiratory complex II was reduced in the mdx52 and mdx52+IT20 groups compared to the WT group (*p* < 0.001 and *p* < 0.001, respectively) (Figure 5F,G). IT100 also increased the levels of mitochondrial respiratory complexes II and III in mdx52 mice (complex II, *p* = 0.002; complex III, *p* = 0.001).

### 3.4 The Phosphorylation Levels of MAPKs Are Not Chronically Elevated After Isometric Interval Training With High and Low Contractile Loads

**FIGURE 6:**
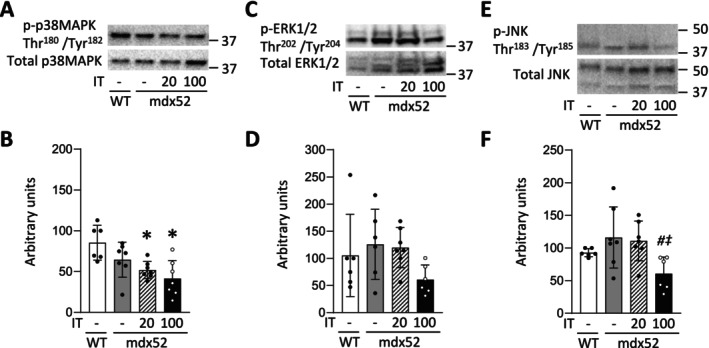
The phosphorylation levels of MAPK are not chronically elevated after isometric interval training with high and low contractile loads. Representative Stain‐Free (S‐F) images and western blots of total and phosphorylated p38 MAPK Thr180/Tyr182 (A), ERK1/2 Thr202/Tyr204 (C), and JNK Thr183/Tyr185 (E) of gastrocnemius muscles in WT and mdx52 mice. The mdx52 mouse underwent isometric interval training with either high (IT100) or low contractile load (IT20). The phosphorylation levels of p38 MAPK Thr180/Tyr182 (B), ERK1/2 Thr202/Tyr204 (D), and JNK Thr183/Tyr185 (F) relative to total protein content. Data show mean ± SD for six to seven muscles per group. One‐way ANOVA with Tukey post hoc test was performed. *p < 0.05 versus WT, ^*#*^ *p* < 0.05 versus mdx52, ^‡^ *p* < 0.05 versus mdx52+IT20.

In Experiment 1, compared to the WT group, the phosphorylation levels of p38 MAPK Thr180/Tyr182, ERK1/2 Thr202/Tyr204, and JNK Thr183/Tyr185 were not altered in the mdx52 group (p38 MAPK Thr180/Tyr182, *p* = 0.242; ERK1/2 Thr202/Tyr204, *p* = 0.180; JNK Thr183/Tyr185, *p* = 1.000) (Figure 6A–F). On the other hand, chronic IT20 and IT100 for 4 weeks reduced the phosphorylation levels of p38 MAPK Thr180/Tyr182 in mdx52 mice compared to the WT group (*p* = 0.024 and *p* = 0.003, respectively). Moreover, chronic IT100, but not IT20, decreased the phosphorylation levels of JNK Thr183/Tyr185 in mdx52 mice (*p* = 0.037 and *p* = 1.000, respectively).

### 3.5 An Acute Bout of Isometric Interval Training With High, but Not Low, Contractile Load Increases the Phosphorylation Levels of p38 MAPK

**FIGURE 7:**
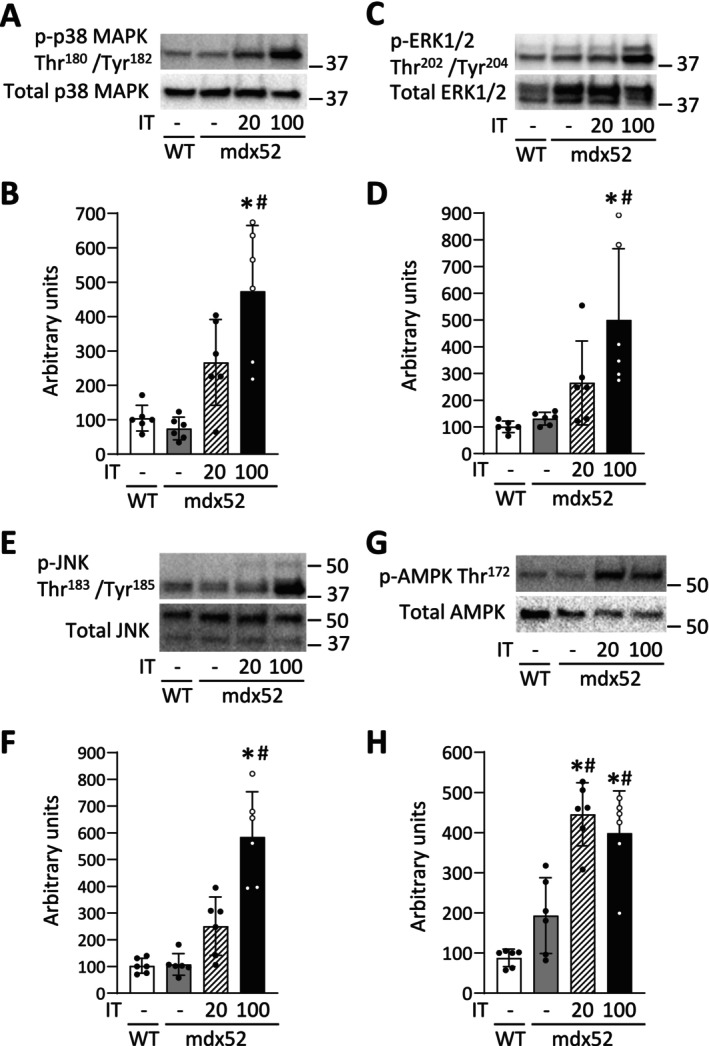
An acute bout of isometric interval training with high, but not low, contractile load increases the phosphorylation levels of p38 MAPK. Representative Stain‐Free (S‐F) images and western blots of total and phosphorylated p38 MAPK Thr180/Tyr182 (A), ERK1/2 Thr202/Tyr204 (C), and JNK Thr183/Tyr185 (E), and AMPK Thr172 (G) of gastrocnemius muscles in WT and mdx52 mice. The mdx52 mouse underwent a single bout of isometric interval training with either high (IT100) or low contractile load (IT20) (Exp 2). The phosphorylation levels of p38 MAPK Thr180/Tyr182 (B), ERK1/2 Thr202/Tyr204 (D), and JNK Thr183/Tyr185 (F), and AMPK Thr172 (H) relative to total protein content. Data show mean ± SD for six muscles per group. One‐way ANOVA with Tukey post hoc test was performed, except for the phosphorylation levels of ERK1/2 Thr202/Tyr204 relative to total protein content, where a Kruskal–Wallis one‐way ANOVA was used on ranks. *p < 0.05 versus WT, ^*#*^ *p* < 0.05 versus mdx52.

In Experiment 2, an acute bout of IT100, but not IT20, significantly increased the phosphorylation levels of p38 MAPK Thr180/Tyr182, ERK1/2 Thr202/Tyr204, and JNK Thr183/Tyr185 in mdx52 mice ([p38 MAPK Thr180/Tyr182] *p* = 0.002 and *p* = 0.068, respectively; [ERK1/2 Thr202/Tyr204] *p* = 0.040 and *p* = 0.637, respectively; [JNK Thr183/Tyr185] *p* = 0.003 and *p* = 0.256, respectively) (Figure 7A–F). In contrast, a single bout of both IT20 and IT100 increased the phosphorylation levels of AMPK Thr172 in mdx52 mice (*p* < 0.001 and *p* = 0.002, respectively) (Figure 7G,H).

**FIGURE 8:**
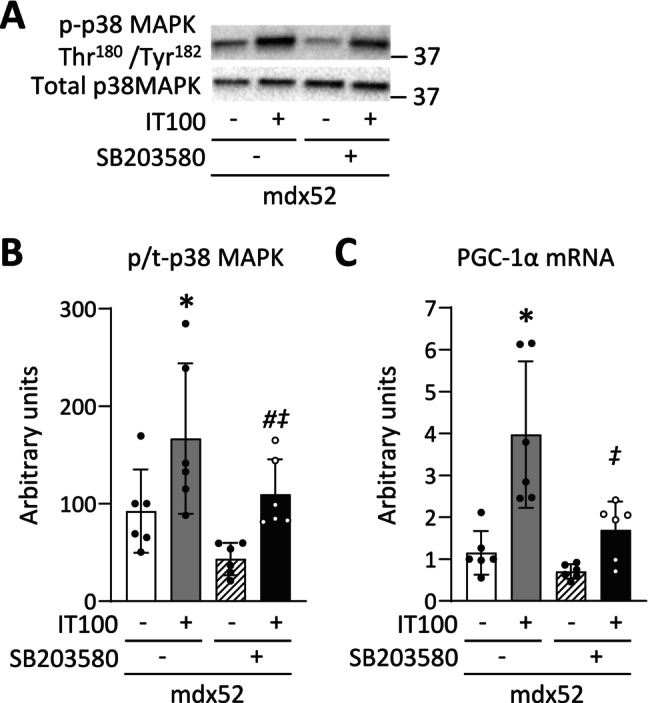
p38 MAPK inhibitor prevents the increase in PGC‐1α mRNA induced by an acute bout of isometric interval training with high contractile load. Representative western blots of total and phosphorylated p38 MAPK Thr180/Tyr182 (A) of gastrocnemius muscles in WT and mdx52 mice. The mdx52 mouse underwent a single bout of isometric interval training with high contractile load (IT100) with or without the administration of SB203580, a p38 MAPK inhibitor (Exp 3). The phosphorylation levels of p38 MAPK Thr180/Tyr182 relative to total protein content (B). The mRNA levels of PGC‐1α (C). Data show mean ± SD for six muscles per group. Two‐way ANOVA with Tukey post hoc test was performed. *p < 0.05 versus mdx52, ^#^ *p* < 0.05 versus mdx52+SB203580, ^‡^ *p* < 0.05 versus mdx52+IT100.

Among the MAPK family, p38 MAPK has been shown to be activated by high‐intensity exercise and to induce PGC‐1α in normal muscle [24]. In line with this, our results show that the transient activation of p38 MAPK by an acute session of IT100 was associated with an increase in PGC‐1α after 4 weeks of IT100 in dystrophin‐deficient muscle. To explore this causal relationship, we conducted mechanistic experiments using SB203580, a p38 MAPK inhibitor. The phosphorylation level of p38 MAPK Thr180/Tyr182 and the mRNA levels of PGC‐1α were increased 180 min after a single bout of IT100 (p38 MAPK Thr180/Tyr182, *p* = 0.013; PGC‐1α, *p* < 0.001) (Figure 8A–C). In contrast, intraperitoneal administration of SB203580 30 min before and immediately after a single bout of IT100 prevented these molecular alterations (p38 MAPK Thr180/Tyr182, *p* = 0.039; PGC‐1α mRNA, *p* < 0.001).

## 4 Discussion

In this study, we examined the effects of isometric IT at varying contractile loads on muscle fatigue resistance and mitochondrial content in young (i.e., 4‐ to 6‐week‐old) mdx52 mice, a period characterized by vigorous muscle necrosis and regeneration [17, 18]. Our data show that isometric IT with high contractile load (IT100), but not low contractile load (IT20), improves fatigue resistance and increases mitochondrial content in dystrophin‐deficient muscles of young mdx52 mice. This was associated with the activation of the p38 MAPK/PGC‐1α pathway. These findings support our hypothesis that isometric IT improves fatigue resistance and mitochondrial content in a contractile load‐dependent manner, even in young mdx52 mice with high disease activity.

Although dystrophic human skeletal muscle undergoes progressive degeneration, the skeletal muscles of mdx mice experience extensive degeneration and regeneration between 2 and 12 weeks of age, but exhibit relatively mild pathology in adulthood [17, 18]. In this study, the percentage of EBD‐positive area, a marker of membrane damage, was approximately 20% in skeletal muscle of young mdx52 mice, which is twice as high as the ~10% observed in adult mdx52 mice in our previous study [14]. Importantly, compared to our previous study using adult mdx52 mice [14], isometric IT100 elicited comparable enhancements in fatigue resistance and mitochondrial content without altering the composition of MyHC isoforms, even in young mdx52 mice. Therefore, isometric IT appears to be a promising approach for increasing mitochondrial content and enhancing fatigue resistance in dystrophin‐deficient muscles. This notion is further supported by studies showing that isometric training safely improves muscle strength in both patients with DMD [12] and mdx mice [13].

We previously demonstrated that the same IT20 protocol conducted in normal mice increases mitochondrial content and fatigue resistance, accompanied by an upregulation of PGC‐1α [16]. However, in this study, these improvements were not observed following IT20 in mdx52 mice. The reason for this discrepancy remains unclear, but it may be attributed to differences in molecular responses to exercise between normal and dystrophic muscle. To explore this, we conducted mechanistic experiments using an acute session of isometric IT, which revealed that p38 MAPK, a key regulatory factor of PGC‐1α responsive to mechanical stress [23, 49], was activated by IT100 but not by IT20 in mdx52 mice. Notably, a single session of IT20 activates p38 MAPK in normal mice (Yamauchi, N., Naito, A., Yamada, T., unpublished observation), suggesting that impaired p38 MAPK activation may be responsible for the blunted PGC‐1α expression observed in mdx52 mice following IT20. Supporting this notion, our data showed that inhibition of p38 MAPK prevented the IT100‐induced increase in PGC‐1α mRNA expression in mdx52 muscles. Thus, these findings suggest that p38 MAPK may serve as a key upstream regulator for the isometric IT‐induced increase in PGC‐1α and, consequently, mitochondrial content in mdx52 mice.

Previous studies investigating the effects of exercise in animal models for DMD have primarily used easily implementable running exercises [50]. However, Nakamura et al. [28] have suggested that wheel running exercise leads to chronic activation of MAPKs, including p38 MAPK, and exacerbates muscle damage in mdx mice. While p38 MAPK is a critical regulator of exercise‐induced cellular adaptations, such as mitochondrial biogenesis [23], its prolonged activation has been linked to the progression and persistence of various pathologies, including DMD [26, 51]. In this study, although we observed a significant increase in p38 MAPK activation immediately after a session of IT100, its basal activity did not increase after 4 weeks of IT100. This may be attributed to the use of low‐damage isometric exercise, which differs from running exercise that includes ECC and is more prone to causing muscle damage. These findings underscore the importance of contraction type as a key factor in determining the efficacy of exercise in DMD.

Intriguingly, we previously observed that IT100 nearly eliminates resting muscle fiber damage in adult mdx52 mice, as indicated by the absence of infiltration of the membrane‐impermeable EBD [14]. Consistent with this, the present study demonstrated that IT100, but not IT20, induces similar beneficial effects even in young mdx52 mice with relatively high disease activity. The reason why only isometric IT100 with high contractile load improved resting muscle damage in dystrophin‐deficient muscle remains to be determined. One possible explanation is that the increase in PGC‐1α expression associated with IT100 plays a role, as previous studies have shown that genetically overexpressing PGC‐1α in dystrophin‐deficient muscle improves resting muscle damage [3, 20, 52].

PGC‐1α has been shown to induce utrophin expression through the N‐box domain in the promoter region of the utrophin gene [53], which is thought to compensate for dystrophin function [54]. However, in this study, IT100 increased PGC‐1α expression and significantly reduced resting muscle damage in dystrophin‐deficient muscle, but this was not accompanied by increased utrophin expression. Supporting this, it has been demonstrated that PGC‐1α overexpression improves dystrophic pathology even in dystrophin–utrophin double‐knockout mice [55]. These findings therefore suggest that PGC‐1α improves dystrophic pathology through a utrophin‐independent pathway [21, 56]. In contrast to our results, however, Hardee et al. [57] reported that utrophin expression is required for mitochondrial adaptations and protection against eccentric contraction‐induced damage in dystrophic muscle following chronic low‐frequency stimulation. The discrepancy between their findings and ours remains unclear, but it may be attributed to the substantially higher training volume used in their study (i.e., 12 h/day for 28 days).

In addition, we observed no changes in the expression levels of other membrane proteins, such as integrin, β‐DG, and α‐SG, which are believed to contribute to membrane stability [58]. Furthermore, although dysferlin, a major membrane repair protein, was elevated in both the IT20 and IT100 groups, histopathological improvements were not observed in the IT20 group. These findings suggest that factors beyond membrane stability and repair capacity may contribute to the improvements in tissue pathology induced by IT100. This highlights the need for further study to better understand the underlying mechanisms.

Despite employing a high contractile load, IT100 did not improve the muscle weakness observed in dystrophin‐deficient muscle. In a previous study, we demonstrated that training with longer contraction durations (2 s contractions) effectively activated the mTOR pathway in a contractile load‐dependent manner, resulting in muscle hypertrophy in rat skeletal muscle [59]. In contrast, the current study utilized shorter contraction durations (0.25 s contractions) to mimic IT. Therefore, it appears that high‐intensity training with short contraction durations may promote muscle adaptation primarily through PGC‐1α pathway, rather than the mTOR pathway.

### 4.1 Limitations

We believe that the findings of this study provide valuable insights for the field of exercise prescriptions for DMD; however, several limitations should be acknowledged. First, our results suggest that IT with high contractile load improves mitochondrial content and muscular fatigue resistance via the p38 MAPK/PGC‐1α pathway; however, these improvements may not rely solely on this pathway. To further clarify its role, pharmacological or genetic inhibition of p38 MAPK and/or PGC1α during chronic IT would be a more direct approach.

Second, although we aimed to adjust the contractile load by using two different stimulation frequencies in our IT protocol, this approach inevitably led to differences not only in contraction intensity but also in the training volume between IT20 and IT100. As a result, it is difficult to determine whether the observed differences in training effects are attributable to contraction intensity, training volume, or a combination of both.

Third, in Experiment 2, we measured MAPK activity after the first IT session, as the activation of these signaling molecules is transient [24], requiring muscle samples to be collected immediately after exercise. In contrast, Experiment 1 aimed to investigate the long‐term effects of training, and MAPK activity was assessed at rest following a sufficient recovery period (i.e., 72 h) after the final IT session. Therefore, it was not feasible to assess acute MAPK activation immediately after the 15th session. In this context, a previous study demonstrated that training‐induced MAPK phosphorylation is blunted after the 10th session compared to the first; however, it remains elevated relative to control groups [60]. Based on this, we considered that although the transient activation of MAPKs may be attenuated after the 15th session compared to the first, a relatively high level of activation was still likely maintained in Experiment 1 of the present study.

Finally, a further limitation is that CS activity was assessed in the plantaris muscle rather than in the gastrocnemius muscle, due to limited tissue availability. While this may affect direct comparability with previous studies focusing on the gastrocnemius, it is worth noting that both muscles are predominantly composed of fast‐glycolytic fibers [61]. Therefore, we believe that the observed adaptations in CS activity are likely representative of changes occurring in both muscles.

## 5 Conclusions

In conclusion, our data show that isometric IT with high, but not low, contractile load improves muscle fatigue resistance in young mdx52 mice with severe dystrophic features. The beneficial effects of isometric IT are likely due to a contractile load‐dependent increase in mitochondrial content, presumably mediated by the transient activation of the p38 MAPK/PGC‐1α pathway in dystrophin‐deficient muscles. Thus, although the capacity for aerobic adaptation is diminished in dystrophin‐deficient muscles compared to normal muscles, it can be improved through isometric IT with high contractile load. However, while the present study employed isometric training with a short contraction duration of 0.25 s, previous studies using longer isometric contraction times have demonstrated acute muscle damage in lumbrical muscles from mdx mice [62, 63]. Therefore, contraction duration may be a crucial factor that determines the benefits and drawbacks of isometric training in dystrophin‐deficient muscles. Further investigation into optimal conditions will be necessary for the successful clinical application of isometric training.

## Author Contributions

Takashi Yamada conceived and designed the research; Nao Yamauchi, Yuki Ashida, Azuma Naito, Nao Tokuda, Ayaka Niibori and Takashi Yamada performed the research and acquired the data. Nao Yamauchi, Norio Motohashi, Yoshitsugu Aoki, and Takashi Yamada analyzed and interpreted the data. The manuscript was drafted by Nao Yamauchi and Takashi Yamada. Takashi Yamada was mainly involved in the revisions, and all authors approved the final, submitted version. All authors agree to be accountable for all aspects of the work. All persons designated as authors qualify for authorship, and all those who qualify for authorship are listed.

## Conflicts of Interest

The authors declare no conflicts of interest.

## Data Availability

The data that support the findings of the study are available on request from the corresponding author.
